# Antecedents of maternal parenting stress: the role of attachment style, prenatal attachment, and dyadic adjustment in first-time mothers

**DOI:** 10.3389/fpsyg.2015.01443

**Published:** 2015-09-24

**Authors:** Claudia Mazzeschi, Chiara Pazzagli, Giulia Radi, Veronica Raspa, Livia Buratta

**Affiliations:** Department of Philosophy, Social and Human Sciences and Education, University of PerugiaPerugia, Italy

**Keywords:** parenting stress, prenatal attachment, first-time mothers, risk factors, dyadic adjustment

## Abstract

The transition to parenthood is widely considered a period of increased vulnerability often accompanied by stress. Abidin conceived parenting stress as referring to specific difficulties in adjusting to the parenting role. Most studies of psychological distress arising from the demands of parenting have investigated the impact of stress on the development of dysfunctional parent–child relationships and on adult and child psychopathology. Studies have largely focused on mothers’ postnatal experience; less attention has been devoted to maternal prenatal characteristics associated with subsequent parental stress and studies of maternal prenatal predictors are few. Furthermore, no studies have examined that association exclusively with samples of first-time mothers. With an observational prospective study design with two time periods, the aim of this study was to investigate the role of mothers’ attachment style, maternal prenatal attachment to the fetus and dyadic adjustment during pregnancy (7th months of gestation) and their potential unique contribution to parenting stress 3 months after childbirth in a sample of nulliparous women. Results showed significant correlations between antenatal measures. Maternal attachment style (especially relationship anxiety) was negatively correlated with prenatal attachment and with dyadic adjustment; positive correlations resulted between prenatal attachment and dyadic adjustment. Each of the investigated variables was also good predictor of parenting stress 3 months after childbirth. Findings suggested how these dimensions could be considered as risk factors in the transition to motherhood and in the very beginning of the emergence of the caregiving system, especially with first-time mothers.

## Introduction

Pregnancy, childbirth and the transition to motherhood involve complex cognitive, affective, and behavioral changes that require restructuring goals, behaviors, and responsibilities to achieve a new conception of self (Mercer, 2004). In addition to experiencing body changes, new mothers undergo the process of attaining their maternal identity (Hung et al., 2011a). The transition to parenthood is widely considered a period of increased vulnerability that is often accompanied by stress (Morse et al., 2000). A mismatch between parents’ perception of the available resources for meeting the demands of parenthood and the perceived demands of the parenting role can cause parental stress (Deater-Deckard et al., 1998).

In particular, Abidin (1995) conceived parenting stress as referring to specific difficulties in adjusting to the parenting role, reflecting parents’ conscious perceptions of their child, their relationship with their child and themselves as parents. Parenting stress, as assessed by the Parenting Stress Index (PSI; Abidin, 1986), one of the most widely used instruments, is considered a factor that influences parenting behavior and a determinant of dysfunctional parenting (Belsky, 1984; Abidin, 1992; Rodgers, 1993).

Most studies of psychological distress arising from the demands of parenting have investigated the impact of stress on the development of dysfunctional parent–child relationships and on adult and child psychopathology (Deater-Deckard et al., 1998). Studies have largely focused on mothers’ postnatal experience (i.e., Ostberg and Hagekull, 2000; Gray et al., 2012); less attention has been devoted to maternal prenatal characteristics associated with the subsequent onset of parental stress.

Studies of maternal prenatal predictors of postpartum parenting stress levels are few. The majority of studies investigate ante-partum depression and anxiety and postnatal parenting stress. Leigh and Milgrom (2008) in a study aiming to identify risk factors predictive of prenatal depression, postnatal depression, and parenting stress, found that prenatal anxiety, prenatal low self esteem and prenatal depression were related to postnatal parenting stress, although the strongest predictor of parenting stress was postnatal depression. Misri et al. (2010), in a study with pregnant women prospectively monitored for depression and anxiety during the third trimester and 3 and 6 months postpartum, found that prenatal depression and anxiety directly impact postnatal maternal parenting stress. In a recent longitudinal birth cohort study following sub-Saharan African women from the third trimester in pregnancy to 2 years postnatal and adjusting for confounders, Guo et al. (2014) found that prenatal depression was associated with parenting stress, while prenatal anxiety was not. Another study focused on the role of a woman’s own parenting history in postnatal parenting stress. Specifically, Grant et al. (2012) examined the associations between perceived parental care and control in childhood assessed during the third trimester of pregnancy and maternal parenting stress at 7 months postpartum. A significant association was found between the maternal perception of parenting as characterized by low care or/and high control and postnatal parenting stress.

Researchers have emphasized the need to examine the relationships among the predictors of parenting stress to develop more comprehensive theoretical models (Abidin, 1992; Ostberg and Hagekull, 2000). Furthermore, no studies have examined the association between postnatal PSI and maternal prenatal characteristics exclusively with samples of first-time mothers. The relevance of studies conducted with nulliparous women arises from research showing that first-time mothers have more difficulties coping with life changes than pluriparous women do (Pridham and Chang, 1989; Cronin and McCarthy, 2003). The transition from the known reality to a new reality that characterizes the experience of the transition to motherhood in nulliparous mothers brings profound changes affecting the reorganization of the self and of the infant’s representations together with transformations in the relationship with one’s partner (Mac Beth-Williams et al., 1987; Lis et al., 2004; Mercer, 2004; Deave et al., 2008).

The mother’s own attachment pattern is considered a powerful predictor of future parenting. The attachment pattern reflects early experiences of handling distress. A secure attachment pattern has been associated with the ability to cope with distress and to adjust to the tasks of parenthood (Alexander et al., 2001; Feeney, 2003; Jones et al., 2015; Pazzagli et al., 2015). Lionetti et al. (2015) found that in the postnatal period attachment state of mind, along with the current experience between partners, contributed to their adjustment to the task of parenting in terms of parenting stress.

Having a baby is a powerful experience that, according to attachment theory, should activate the attachment system and the related behavioral systems, such as caregiving behavior (Bowlby, 1988). The transition to motherhood requires a new organization of mental life that is adapted to the reality of caring for an infant (Stern, 1998). The development of a tie between the mother and her fetus has been conceptualized and assessed as antenatal attachment. Specifically, Condon (1993) conceived the term “parent-to-infant attachment”, to refer to the emotional bond or tie of affection experienced by the parent toward the infant (Condon and Corkindale, 1998). So, a key component of the construct seems to be the protection for the fetus, expressed by maternal disposition toward the fetus of knowing, being with, protecting, gratifying needs, and avoiding loss (Condon, 1993; Walsh et al., 2014). Recent contributions have proposed that this bond, which involves attending to needs and providing protection, is indicative not of the attachment system but of the caregiving system (George and Solomon, 1999; Brandon et al., 2009; Walsh et al., 2014). During pregnancy, antenatal attachment to the fetus is associated with several maternal characteristics, such as attachment style and the quality of the relationship with one’s partner (Condon and Corkindale, 1997; Bloom, 1998; Mikulincer and Florian, 1999; White et al., 1999; Barone et al., 2014; Walsh et al., 2014). In the postpartum period, studies have found associations between antenatal attachment and familial and parental functioning, such as the quality of mother–infant interaction and child attachment (Siddiqui and Hagglof, 2000; Cannella, 2005; Alhusen, 2008; Crawford and Benoit, 2009).

The transition to parenthood also requires adaptive changes in the couple’s relationship (Hazan and Shaver, 1994). As observed by Durkin et al. (2001), if prospective parents feel emotionally distant from and unsupported by their partners, then their adjustment to parenthood is likely to be negatively affected. Studies show that family functioning contributes to parenting satisfaction and perceptions of negative marital quality is associated with higher degrees of parenting stress (Horowitz and Damato, 1999; Ostberg and Hagekull, 2000; Salonen et al., 2010). In particular, in a longitudinal study with a sample of first-time parents, Morse et al. (2000) found that poor relationship functioning at mid-pregnancy predicted vulnerability to postnatal distress. The quality of dyadic adjustment during the transition to parenthood has also been associated with attachment patterns (Paley et al., 2005; Velotti et al., 2011; Parker et al., 2013).

On the basis of the paucity of studies on maternal prenatal predictors of postnatal parenting stress and the lack of research conducted exclusively with first-time mothers, the aims of the present study were:

(1) to investigate the role of certain psychological aspects of maternal functioning during the transition to motherhood in a sample of first-time mothers;
(2) to assess their unique role in maternal postnatal adjustment at the very beginning of the relationship with the newborn.

Specifically, using an observational prospective study with two time periods (pre – and postnatal), this paper explored the following in a group of nulliparous women:

(1) the associations between maternal attachment style, maternal antenatal attachment to the fetus (MAAS) and dyadic adjustment in the prenatal period;
(2) how maternal parenting stress, as assessed 3 months after delivery, can be predicted by each of the prenatal variables investigated.

On the basis of the reported studies, a relationship among the mother’s attachment pattern, the emotional bond with the fetus and the quality of the relationship with the partner was expected. Furthermore, a prediction of each of these prenatal variables of the onset of postnatal maternal parenting stress was hypothesized.

## Materials and Methods

### Procedure and Participants

An observational prospective study focused on the investigation of certain psychological factors contributing to the construction of the caregiving system in nulliparous women and their power in predicting parenting stress with two time periods, before the child’s birth, – at the 7th months of gestation (32nd weeks) – and 3 months after the child’s birth was used.

The study was approved by the University of Perugia Ethics Committee and was conducted in accordance with the Helsinki Declaration.

Participants were enrolled at the Operative Unit of Obstetrics and Gynecology (OUOG) of a hospital in central Italy. The choice of the hospital resulted from the availability of the unit staff to participate in the study. Pregnant women in this unit received antenatal care from obstetricians. During childbirth classes, obstetricians invited pregnant women to participate in the study, providing a preliminary information session on the aims and two-step methodology of the research. The obstetricians received preliminary training by a study researcher to make them feel confident in responding to any women’s questions about the study and its procedures.

For the enrollment, because of the use of questionnaires, the inclusion criteria required the women to be able to read and understand Italian; furthermore, because of the specificity of the questionnaire regarding the couple relationship, the women needed to have a romantic partner and to be in the 32nd weeks of gestation (plus or minus 1 week). This point of pregnancy was chosen because of the evidence of increased expectations regarding the unborn child during this phase of pregnancy as well as because of the increase of fetal movements (Lis et al., 2004).

After this presentation, the women were given a packet that included the consent form to participate to the first phase of the study and a consent form to be contacted for the second phase. At approximately the 32nd weeks of gestation, the women who had given written informed consent completed the prenatal measures (including a questionnaire on socio-demographic information) at the hospital before the beginning of a childbirth class. The time needed to complete the questionnaires in the first phase was approximately 50 min. Approximately 3 months after their children’s birth, the women who had given consent to participate in the second phase of the study, were invited by phone to return to the hospital to complete the postnatal questionnaire. The time needed for this second phase was approximately 20 min.

A total of 130 packets were distributed; 95 (73%) women agreed to participate in both phases of the study; 5 (5%) questionnaires were not fully complete at the first step and were then excluded; and of the 90 (95%) women who agreed to participate, 20 (22%) refused to participate in the second phase when contacted because they could not reach the hospital. Only women who had completed both the first and second phase measures were included in the data analysis.

### Sample

The total sample consisted of 70 women recruited at the OUOG of a hospital in central Italy. All the women were nulliparous and were in the 32nd weeks of gestation (plus or minus 1 week). The mean age of the women was 32.75 (*SD* = 4.84); they belonged to a medium socio-economic status (SES) level (Mean = 36.67; *SD* = 12.26). Other socio-demographic information are reported in **Table 1**.

## Measures

### Prenatal Measures

#### Socio-Demographic Questionnaire

This series of questions included the participants’ age, level of education, employment status, family structure and the duration of the marital/conjugal relationship. Familial SES was calculated using the Hollingshead Index of Social Position (Hollingshead, unpublished manuscript).

#### Attachment Style Questionnaire (ASQ; Feeney et al., 1994)

The ASQ is a 40-item Likert-type self-report questionnaire designed to measure five dimensions of adult attachment that are central to Hazan and Shaver’s (1987) and Bartholomew’s (1990) conceptualizations of attachment: Confidence in Self and Others (eight items), Discomfort with Closeness (10 items), the Need for Approval (seven items), Preoccupation with Relationships (eight items), and Relationships as Secondary (sevan items). Each item is rated on a 6-point scale ranging from 1 (“totally disagree”) to 6 (“totally agree”). The ASQ has shown adequate reliability (Feeney et al., 1994; Fossati et al., 2003), with Cronbach’s alpha coefficients for the five scales ranging from 0.81 to 0.87. According to previous studies (Alexander et al., 2001; Feeney, 2003), two major factors were computed for these scales.

#### Discomfort with Closeness and Relationship Anxiety

The former (16 items) measures the tendency to be uneasy with intimacy and dependency in relationships; the latter (15 items) measures concerns about the attachment other’s feelings of love and fears of being rejected. In the present study, the Italian version of this measure was used (Fossati et al., 2007), with internal consistency in terms of Cronbach’s alpha coefficients of 0.80 and 0.85 for the two major factors.

#### Maternal Antenatal Attachment Scale (MAAS; Condon, 1993)

Developed to measure the emotional bond between a pregnant woman and her unborn child, the MAAS consists of 19 items focused on the past 2 weeks rated on a 5-point scale. Two scales Attachment Quality (AQ) and Attachment Intensity (AI) are combined to obtain a total score. The two scales, respectively, measure the quality of the emotional bond AQ and the time spent in attachment mode (the intensity of preoccupation). The internal consistency of the instrument is acceptable (Condon, 1993). In the present study, the Italian version of the MAAS was used (Righetti et al., 2005), with an internal consistency in terms of Cronbach’s alpha of 0.81 for the total score, of 0.80 for AQ scale, and of 0.82 for AI scale.

#### Dyadic Adjustment Scale (DAS; Spanier, 1976)

Developed to measure conjugal adjustment, the DAS can be used with married or unmarried couples engaged in a dyadic romantic relationship. Consisting of 32 items, the scale has a range of scores from 0 to 151. Lower scores indicate distress and divergence in the dyadic relationship. The DAS has shown adequate psychometric properties and good internal consistency (α = 0.95; Carey et al., 1993). In the present study, the Italian validated version of the DAS was used (Gentili et al., 2002). The internal consistency coefficient in terms of Cronbach’s alpha for the total score was 0.85.

### Postnatal Measure

#### Parenting Stress Index: (PSI-SF; Abidin, 1986)

This self-report scale was developed to measure stress associated with the parenting role and is used as a screening instrument for dysfunctional parenting. It consists of 36 items rated on a 5-point scale indicating the degree to which each item has been a problem for the parent during the past week. The composite total score range is between 36 and 158, where lower scores indicate lower overall levels of parenting stress. The PSI-SF has shown good overall psychometric properties (Abidin, 1986). The internal consistency reliability for the composite total score was reported by the author to be 0.91. The Italian validated version of the PSI-SF (Guarino et al., 2008) was used in this study. The internal consistency coefficient for the present sample was 0.89 for the total score.

## Data Analysis

Standard descriptive statistics in the form of means and standard deviations or frequencies and percentages were used to summarize the sample’s socio-demographic characteristics and used in the measures assessed. A series of one-sample Kolmogorov–Smirnov tests (Z), were conducted and showed that the measures were distributed normally. Pearson’s correlation analyses were conducted on the prenatal measures to examine the associations between maternal attachment style, MAAS and dyadic adjustment. *p*-values of 0.05 or less were first identified as statistically significant; Bonferroni’s correction was applied, with critical alpha value set to 0.001 (0.005/25 correlations). The effect size of the correlation was classified according to Cohen (1992): low effect size ≤0.30; medium effect size = 0.31–0.50; and large effect size ≥0.50. To measure the single contribution of each of the prenatal measures to the prediction of postnatal parenting stress, a regression analysis was conducted. Because of the paucity of the studies on maternal prenatal characteristics associated with the subsequent parental stress, the regression analysis was conducted separately for each of the variable investigated, in order to test the existence of each variable’s unique effect, as a first investigation of their specific contribution. In the first and the second model two Multivariable Linear Regressions were performed separately in order to test the effect of the two factors of maternal attachment (Discomfort with Closeness and Relationship Anxiety) and of the maternal antenatal attachment AQ and AI. A Univariate Regression was also performed for testing the effect of conjugal adjustment. The data were analyzed using SPSS version 21.0.

## Results

Descriptive statistics for the sample socio-demographic characteristics are reported in **Table 1**.

**Table 1:** Descriptive statistics for the socio-demographic characteristics.

	*M*	*SD*
**Socio-demographic variables**		
Age	32.75	4.84
SES	36.67	12.26
Time length of the couple relationship (years)	3.15	1.48
**Family situation**	***N***	**%**
Married and cohabiting	45	64.3
Unmarried and cohabiting	25	35.7


Means (*M*) and standard deviations of the pre-natal and post-natal measures are reported in **Table 2**.

**Table 2:** Descriptive statistics for the antenatal and postnatal measures.

Pre-natal measures	*M*	*SD*	Range	Z	*p*
ASQ – DC	48.89	8.41	28–68	0.134	0.200
ASQ – RA	41.67	9.03	17–61	0.147	0.200
MAAS – TOT	78.74	4.62	66–88	0.198	0.117
MAAS – AQ	46.78	2.28	39–50	0.305	0.112
MAAS – AI	30.28	3.46	23–37	0.305	0.200
DAS TOT	121.84	11.59	75–146	0.186	0.321
**Post-natal measure**					
PSI-SF, TOT	60.48	14.54	36–101	0.169	0.212


*ASQ – DC, Attachment Style Questionnaire, Discomfort with Closeness; ASQ – RA, Attachment Style Questionnaire, Relationship Anxiety; MAAS – TOT, Maternal Antenatal Attachment, Total Score; MAAS – AQ, Maternal Antenatal Attachment, Attachment Quality; MAAS – AI, Maternal Antenatal Attachment, Attachment Intensity; DAS TOT, Dyadic Adjustment Scale Total Score; PSI – SF, TOT, Parenting Stress Index – Short Form, Total Score.*

Regarding MAAS, data were compared with a previous study conducted with women at 19–23 weeks of gestation in terms of confidence interval. Previous data revealed a slightly lower mean total score (*M* = 75.7, *SD* = 8.15), 95% CI (73.29, 78.11) than that obtained for this sample (Righetti et al., 2005). This was perhaps due to the specific time of measure in the present study: the last trimester of pregnancy is a time that is particularly rich in emotions and fantasies because of the increase in fetal movements (Ammaniti et al., 1992; Lis et al., 2004). The dyadic adjustment score for this group had a mean value of 121.84 (*SD* = 11.59). The data were consistent with previous Italian data on nulliparous women (Pazzagli et al., 2015). With regard to postnatal parenting stress, the mothers in the sample showed a mean value of 60.48 (*SD* = 14.54). Compared with the normative data for the age subgroup (1 month/2 years and 11 months) extracted from the total sample of the Italian validated version of the scale, the mean score for the present sample showed a lower value than the normative Italian sample (*M* = 69.27, *SD* = 16.91); 95% CI (68.13, 70.42; Guarino et al., 2008).

### Prenatal Measures

The correlation analysis revealed the existence of significant associations between the antenatal measures after Bonferroni’s correction was applied (**Table 3**). According with Cohen’s criteria (Cohen, 1992), the effect sizes for the significantly correlated measures were generally moderate to large. The correlations between mothers’ attachment style (ASQ) and MAAS revealed the existence of a significant small negative association of Relationship Anxiety with the MAAS – AQ score (*r* = -0.361, *p* = 0.001). Relationship Anxiety was also negatively correlated with dyadic adjustment (DAS TOT; *r* = -0.485, *p* < 0.001). The total score for dyadic adjustment (DAS TOT) was strongly and positively correlated with MAAS – TOT (*r* = 0.447, *p* < 0.001) and with AQ (*r* = 0.528, *p* < 0.001). The smaller low correlation for the AI subscale (*r* = 0.256, *p* = 0.033) was not significant after Bonferroni’s correction.

**Table 3:** Correlations between antenatal measures.

	MAAS – TOT	MAAS – AQ	MAAS – AI	DAS TOT
ASQ – DC	-0.189	-0.197	-0.127	-0.350^∗^
ASQ – RA	-0.256	-0.361^∗^	-0.104	-0.485^∗^
MAAS – TOT	-	0.760^∗^	0.900^∗^	0.;447^∗^
MAAS – AQ		-	0.425^∗^	0.528^∗^
MAAS – AI			-	0.256


*ASQ – DC, Attachment Style Questionnaire, Discomfort with Closeness; ASQ – RA, Attachment Style Questionnaire, Relationship Anxiety; MAAS – TOT, Maternal Antenatal Attachment, Total Score; MAAS – AQ, Maternal Antenatal Attachment, AQ; MAAS – AI, Maternal Antenatal Attachment, Attachment Intensity; DAS TOT, Dyadic Adjustment Scale Total Score. ^∗^p < 0.001 [Bonferroni correction (0.05/25 correlations; N = 70].*

### Prediction of Postnatal Stress

Results of the regression analysis showed that mother’s attachment style explained 38.1% of the variance (*R*^2^ = 0.381, *F*(2, 68) = 3.699, *p* = 0.047). In detail, Relationship Anxiety (β = 0.516, *SE* = 0.496, *t* = 2.271, *p* = 0.041) significantly predicted postnatal parenting stress, whereas Discomfort with Closeness (β = 0.345, *SE* = 0.351, *t* = 1.519, *p* = 0.155) did not.

Maternal antenatal attachment accounted for 44.5% of the variance in parenting stress (*R*^2^ = 0.445), which was significant [*F*(2, 68) = 4.807, *p* = 0.029]. AQ (β = -0.737, SE = 1.898, *t* = -3.094, *p* = 0.009) emerged as a significant predictor for postnatal parenting stress, while AI (β = 0.274, *SE* = 1.142, *t* = 1.149, *p* = 0.879) was not.

Total dyadic adjustment was a significant negative predictor of postnatal maternal parenting stress (β = -0.435, SE = 0.123, *t* = -4.402, *p* < 0.001) accounting for 18.9% of variance in maternal parenting distress [*F*(1,69) = 19.380, *p* < 0.001]. Results are reported in **Table 4**.

**Table 4:** Results of regression analyses for antenatal measures on the Parenting Stress Index, Total Score, at 3 months after childbirth.

		*R*	*R*^2^	β	*SE*	*T*	*p*
Model 1		0.618	0.381	-0.14.69		-0.528	0.042^∗^
	ASQ – Discomfort			0.534	0.351	1.519	0.155
	ASQ – Anxiety			1.127	0.496	2.271	0.041^∗^
Model 2		0.667	0.445			3.684	0.023^∗^
	MAAS – AQ			-0.737	1.898	-3.094	0.009^∗∗^
	MAAS – AI			0.274	1.142	-0.155	0.879
Model 3	DAS TOT	0.435	0.189	-0.435	0.123	-4.402	0.001^∗∗∗^


*ASQ – DC, Attachment Style Questionnaire, Discomfort with Closeness; ASQ – RA, Attachment Style Questionnaire, Relationship Anxiety; MAAS – TOT, Maternal Antenatal Attachment, Total Score; MAAS – AQ, Maternal Antenatal Attachment, AQ; MAAS – AI, Maternal Antenatal Attachment, Attachment Intensity; DAS TOT, Dyadic Adjustment Scale Total Score; PSI – SF, Parenting Stress Index – Short Form, Total Score (^∗^*p* < 0.05, ^∗∗^p < 0.01, ^∗∗∗^p < 0.001).*

## Discussion

This study contributes to the research on parenting in first-time mothers by investigating the relationships between some psychological aspects of maternal functioning in late pregnancy and their potential contribution to predict parenting stress during the early period of the mother–child relationship, after childbirth. Because, as noted previously, few empirical papers have addressed this issue, the present paper provides a contribution to the field of research devoted to identifying the contribution of prenatal variables to the onset of postnatal maternal parenting stress. Furthermore, this paper, which focuses on a sample of nulliparous women, contributes new data to a field of research that currently lacks information on this population.

Specifically pertaining to the first aim, this study examined the maternal attachment style, the maternal antenatal bond to the fetus and dyadic adjustment as prenatal factors, and the data showed interesting connections. The literature on parenting states that differences in attachment have implications for the transition to parenthood, with a positive link between attachment security and parenting (Rholes et al., 1995; Mikulincer and Florian, 1999). Conversely, attachment insecurity has been conceived as a risk factor in the development of parenting. Consistent with previous studies, particularly that of Grant (Grant et al., 2012), the findings of this paper showed the importance of attachment to others in adulthood during childhood in the transition to parenthood.

According to Feeney (2003), this study focused on two dimensions of attachment insecurity: discomfort with closeness and relationship anxiety, which, respectively, measure the tendency to be uneasy with intimacy and concerns about the attachment other’s feelings of love, alongside fears of being rejected. Attachment anxiety was negatively correlated with maternal antenatal attachment, especially to the quality of the emotional bond, and to the adjustment in the couple relationship. Mothers-to-be who were found to be more anxious and preoccupied in their personal attachment have also less positive feelings about the fetus and were less confident in their relationship with their partner. The results of the present study appear to be consistent with previous studies showing such associations (Condon and Corkindale, 1997; Bloom, 1998; White et al., 1999; Barone et al., 2014; Walsh et al., 2014), especially studies devoted to exploring the link between adult attachment patterns and antenatal attachment. Moreover, this research contributes data on the relationship between antenatal attachment to the fetus and couple relationships, a field that has been little investigated by the previous literature (Alhusen, 2008). Antenatal attachment, previously explored as aspect connected to attachment system (Cannella, 2005), has recently been viewed as a bond that involves attending to the child’s needs and providing protection. Recent studies have suggested considering antenatal attachment to be indicative of the caregiving system instead of (only) the attachment system (Brandon et al., 2009; Walsh et al., 2014) and thus to be particularly informative of the parental challenges connected to the transition to parenthood (George and Solomon, 1999). The couple relationship also faces challenge in the transition to parenthood because of the many re-organizations that this moment brings for the couple: in the partners’ relationship quality, in their responsibilities and in their reciprocal routines (Antonucci and Mikus, 1988; Hazan and Shaver, 1994). Particularly, the birth of the first child has been found to have detrimental effects on partnerships (Cowan et al., 1985; Belsky and Rovine, 1990), and relationship quality can decrease as a result of the decline in marital satisfaction and feelings of love that can result from the challenging new task of caring for a newborn.

Based on the need to develop more comprehensive theoretical models to explore the relationships among the predictors of parenting stress (Abidin, 1992), the second aim of this study used a measure at 3 months after the child birth to show that each of the investigated variables was a good predictor of parenting stress 3 months after childbirth. In particular, the mother’s adult attachment, specifically the anxiety dimension, contributed to this predictive power. These results are consistent with findings indicating the implications of differences in attachment for parenting (Jones et al., 2015). Secure mothers have less difficulty coping with parenthood tasks and are more capable in engaging in supportive parenting behavior. Conversely, in a paper studying the effect of child care on infant development, the authors found that parenting stress was significantly associated with insecure (child) attachment to mothers and fathers (Jarvis and Creasey, 1992). While attachment security can be considered a protective factor against parenting stress, attachment insecurity can be considered a risk factor in predicting parenting stress.

Moreover, maternal antenatal attachment to the fetus significantly predicted parenting stress 3 months postpartum, with the specific contribution of AQ. Previous findings that were largely focused on the association between these dimensions were not consistent, with some studies indicating an inverse correlation between stress and antenatal attachment and others unable to replicate such results (Cranley, 1981; Curry, 1987; Grace, 1989). Moreover, findings from the literature suggest that the correlation is also linked to the specificity of the postnatal period investigated. Our data are consistent with the few studies that have investigated this association from a longitudinal perspective (Alhusen, 2008). The findings of this study are consistent with those of Mikulincer, who found such a relationship during the first trimester (Mikulincer and Florian, 1999). According to our data, negative AQ during pregnancy seems to be a risk factor in predicting parenting stress during the first 3 months after childbirth. These findings contribute to filling the gap in evidence in the literature (Alhusen, 2008).

Regarding dyadic adjustment, findings showed its predictive role. According to the present data, a low level of dyadic adjustment during pregnancy was a risk factor for the onset of parenting stress during the initial period after childbirth. Previous data showed that if a woman lacks adequate social support during pregnancy, the result would be negative outcomes, such as postpartum depression and insensitive parenting behavior (Crockenberg, 1981; Cutrona, 1984). Conversely, women who receive support during pregnancy have more positive physical and mental health outcomes during the postnatal period (Collins et al., 1993). Moreover, Hung et al. (2011b) found that women with longer marriages had a significantly lower level of postpartum stress. According to Krieg (2007), being in a marital relationship buffers stress during the postpartum transition. Mothers with a longer length of marriage generally experience more positive qualities in their marriages, with decreased role differentiation and increased role satisfaction in the transition to motherhood (Knauth, 2001).

As noted previously in this paper, parenting stress is a construct that can be affected by different types of stressor. Parenting stress can be considered a function of many different variables (Crnic and Low, 2002). In this paper, the role of some of these variables was considered, but the results need to be interpreted with caution because the relative influence of the different sets of variables were not considered together in the same model. Moreover, the proposed models are not exhaustive. This is a another limitation of the study because other dimensions could affect and mediate the connections observed; for example, everyday tasks specifically associated with parenting as well as the role of children’s characteristics (e.g., temperament) should be added to the model to increase understanding of the phenomenon. Notably, this sample of first-time mothers showed lower levels of parenting stress with respect to normative data (thus, low level of parenting stress) and it should be important to further investigate such connection with a larger first-time mothers sample. Moreover, this paper did not consider the specific role played by the women’s partner. Another limitation of the study is the exclusive use of a self-report procedure to investigate both prenatal variables and postnatal parenting stress. Further studies should incorporate other psychological aspects, including also observational procedure, to obtain a richer picture of the dimensions that affect the initial emergence of the caregiving system and its first challenges, especially with first-time mothers. In spite of these limitations, findings from this study suggest how important is the role of prenatal characteristics in first-time mothers and underline the need to be considered in order to develop effective prevention plans.

## Conflict of Interest Statement

The authors declare that the research was conducted in the absence of any commercial or financial relationships that could be construed as a potential conflict of interest.
